# Synthesis and Evaluation of ^99m^Tc(CO)_3_ Complexes with Ciprofloxacin Dithiocarbamate for Infection Imaging

**DOI:** 10.3390/pharmaceutics16091210

**Published:** 2024-09-14

**Authors:** Afroditi Papasavva, Nektarios N. Pirmettis, Antonio Shegani, Eleni Papadopoulou, Christos Kiritsis, Maria Georgoutsou-Spyridonos, Dimitrios C. Mastellos, Aristeidis Chiotellis, Patricia Kyprianidou, Maria Pelecanou, Minas Papadopoulos, Ioannis Pirmettis

**Affiliations:** 1 Institute of Nuclear and Radiological Sciences and Technology, Energy & Safety, NCSR “Demokritos”, 15310 Athens, Greece; papasavvaphro@gmail.com (A.P.); nnpirmettis@pharm.uoa.gr (N.N.P.); ant.she@hotmail.gr (A.S.); el.papadopoulou@gmail.com (E.P.); kiritsis.chr@gmail.com (C.K.); maria.georgoutsou.spyridonos@gmail.com (M.G.-S.); mastellos@rrp.demokritos.gr (D.C.M.); achiotel@rrp.demokritos.gr (A.C.); pkypri@rrp.demokritos.gr (P.K.); mspap@rrp.demokritos.gr (M.P.); 2 Institute of Biosciences & Applications, NCSR “Demokritos”, 15310 Athens, Greece; pelmar@bio.demokritos.gr

**Keywords:** ciprofloxacin, technetium-99m, tricarbonyl, infection

## Abstract

**Background:** The accurate diagnosis of bacterial infections remains a critical challenge in clinical practice. Traditional imaging modalities like computed tomography (CT) and magnetic resonance imaging (MRI) often fail to distinguish bacterial infections from sterile inflammation. Nuclear medicine, such as technetium-99m (^99m^Tc) radiopharmaceuticals, offers a promising alternative due to its ideal characteristics. **Methods:** This study explores the development of [2 + 1] mixed-ligand ^99m^Tc-labeled ciprofloxacin dithiocarbamate (Cip-DTC) complexes combined with various phosphine ligands, including triphenylphosphine (PPh_3_), tris(4-methoxyphenyl)phosphine (TMPP), methyl(diphenyl)phosphine (MePPh_2_), dimethylphenylphosphine (DMPP), and 1,3,5-triaza-7-phosphaadamantane (ADAP). The characterization of ^99m^Tc-complexes was conducted using rhenium analogs as structural models to ensure similar coordination. **Results:** Stability studies demonstrated the high integrity (97–98%) of the complexes under various conditions, including cysteine and histidine challenges. Lipophilicity studies indicated that complexes with higher logD_7.4_ values (1.6–2.7) exhibited enhanced tissue penetration and prolonged circulation. Biodistribution studies in Swiss Albino mice with induced infections and aseptic inflammation revealed distinct patterns. Specifically, the complex *fac*-[^99m^Tc(CO)_3_(Cip-DTC)(PPh_3_)] (**2′**) showed high infected/normal muscle ratios (4.62 at 120 min), while the complex *fac*-[^99m^Tc(CO)_3_(Cip-DTC)(TMPP)] (**3′**) demonstrated delayed but effective targeting (infected/normal muscle ratio of 3.32 at 120 min). **Conclusions:** These findings highlight the potential of ^99m^Tc-labeled complexes as effective radiopharmaceuticals for the differential diagnosis of bacterial infections, advancing nuclear medicine diagnostics. Future studies will focus on optimizing molecular weight, lipophilicity, and stability to further enhance the diagnostic specificity and clinical utility of these radiopharmaceuticals.

## 1. Introduction

The accurate diagnosis of infections remains a critical challenge in clinical practice, particularly in differentiating bacterial infections from sterile inflammation [1]. Traditional imaging modalities, such as computed tomography (CT) and magnetic resonance imaging (MRI), often fail to distinguish between these conditions effectively [2]. Nuclear medicine, leveraging the unique properties of radiopharmaceuticals, offers a promising alternative. Among these, technetium-99m (^99m^Tc) is the radionuclide of choice due to its ideal characteristics, including its short half-life (6.02 h), emission of 143 keV gamma rays, low energy conversion and Auger electrons, absence of β-emission, low cost, and availability from a ^99^Mo/^99m^Tc generator. These properties make ^99m^Tc an attractive candidate for developing targeted radiopharmaceuticals [3].

In nuclear medicine, the gold standard for infection imaging has long been the use of radiolabeled white blood cells (WBCs) using Single Photon Emission Computed Tomography (SPECT) [4]. This technique is highly specific for detecting infections, as it relies on the natural migration of labeled leukocytes to sites of infection. Despite its effectiveness, WBC SPECT is labor-intensive, requires blood handling, and may pose contamination risks. Furthermore, the sensitivity of WBC SPECT can be limited, particularly in chronic infections where leukocyte recruitment is less pronounced [5].

While Positron Emission Tomography (PET) has emerged as a complementary modality in nuclear medicine, offering higher sensitivity and resolution, its application in infection imaging is still evolving [6]. PET tracers, such as ^18^F-FDG, are commonly used but lack specificity as they accumulate in inflammation sites. Recent developments in ^18^F-labeled antibiotics, such as ^18^F-ciprofloxacin, have shown the potential to improve specificity by targeting bacterial infections. However, SPECT, particularly with ^99m^Tc-labeled compounds, remains a more accessible and cost-effective option in many clinical settings, particularly for established protocols in infection imaging [7].

Recent developments in SPECT radiotracers, such as 99mTc-labeled antibiotics as specific infection imaging agents, have gained significant interest. Ciprofloxacin, a broad-spectrum fluoroquinolone antibiotic, has been extensively studied for this purpose [8,9,10,11,12,13]. Its mechanism of action involves the inhibition of bacterial DNA replication by binding to DNA gyrase and topoisomerase IV, making it an attractive candidate for radiolabeling [14]. The first ^99m^Tc-labeled ciprofloxacin derivative, known as Infecton™, demonstrated promising results in early clinical trials, with overall sensitivity and specificity values of 88% and 82%, respectively [15,16,17].

Despite these initial successes, subsequent studies revealed inconsistencies in the binding specificity of ^99m^Tc-ciprofloxacin to bacteria, with conflicting results regarding its uptake in tissues with aseptic inflammation [18,19]. These variations were attributed, in part, to the lack of structural characterization of the ^99m^Tc-fluoroquinolone complexes formed [20]. This underscored the need for more defined and stable radiopharmaceuticals. Recent advancements have focused on improving the stability and specificity of ^99m^Tc-labeled ciprofloxacin derivatives. One approach involves the use of ciprofloxacin dithiocarbamate (Cip-DTC), which combines the antibiotic properties of ciprofloxacin with the chelating ability of dithiocarbamate groups [21,22,23]. Studies have shown that Cip-DTC labeled with both the nitrido core [22] [^99m^Tc≡N]^2+^ and the ^99m^Tc-tricarbonyl [23] precursor *fac*-[^99m^Tc(CO)_3_(H_2_O)_3_]^+^ exhibited higher accumulations in infected tissues in animal models.

To further enhance the specificity and stability of these complexes, we explored the [2 + 1] mixed-ligand strategy, combining Cip-DTC with various phosphine ligands [24,25]. Phosphines are versatile ligands known for their ability to form stable bonds with metal centers [26,27,28]. This strategy aims to produce well-defined, neutral tricarbonyl complexes with improved biodistribution and targeting properties. In this paper, we report the synthesis, characterization, and preliminary biological evaluation of new [2 + 1] mixed-ligand ^99m^Tc-tricarbonyl complexes of Cip-DTC and various phosphine derivatives. The dithiocarbamate group acted as a bifunctional chelator, coordinating to the metal while retaining the biological activity of ciprofloxacin. The phosphine ligands, including triphenylphosphine (PPh_3_), tris(4-methoxyphenyl) phosphine (TMPP), methyl(diphenyl)phosphine (MePPh_2_), dimethylphenylphosphine (DMPP), and 1,3,5-triaza-7-phosphaadamantane (ADAP), provided additional stability and specificity through their coordination to the *fac*-[M(CO)_3_]^+^ core. The synthesis of rhenium complexes as structural models for the corresponding ^99m^Tc-complexes ensured that the analogous ^99m^Tc complexes displayed similar coordination parameters and physical properties. This facilitated the structural characterization of the ^99m^Tc-complexes, enabling us to draw parallels between the rhenium and technetium systems. Through this approach, we aimed to develop effective infection imaging agents with high specificity and stability, advancing the field of nuclear medicine diagnostics.

## 2. Experimental Section

### 2.1. Materials and Methods

All reagents and organic solvents used in this study were of reagent grade and used without further purification. Flash chromatography was performed using Fluka silica gel. Solvents for high-performance liquid chromatography (HPLC) were HPLC-grade, filtered through 0.22 μm membrane filters (Millipore, Milford, MA, USA), and degassed by a helium flux before and during use. Infrared (IR) spectra were recorded on a Bruker Alpha II FT-IR/ATR spectrometer (Bruker, Billerica, MA, USA) in the 4000–400 cm^−1^ range. Nuclear magnetic resonance (NMR) spectra were recorded on a Bruker 500 MHz Advance DRX spectrometer (Bruker, Billerica, MA, USA) using tetramethylsilane ((CH_3_)_4_Si) as an internal reference. HPLC analysis was performed on a Thermo Scientific Dionex UltiMate 3000 series system coupled with a photodiode array detector and an Eckert & Ziegler HPLC Scan gamma detector (NaI). Separations were achieved on an EC Nucleosil 5 μm C18 100 Å 150 × 4 mm column eluted with a binary gradient system at a flow rate of 1 mL·min^−1^. Mobile phase A consisted of water with 0.1% trifluoroacetic acid (TFA), while mobile phase B consisted of methanol with 0.1% TFA. The elution profile started with 5% B and 95% A for 1 min, followed by a linear gradient to 90% B over 10 min. This gradient was maintained at 90% B for 10 min, then returned to 5% B over 5 min and remained at 5% B for 5 min before the next injection. The ciprofloxacin dithiocarbamate (Cip-DTC) [29], rhenium pentacarbonyl, and tricarbonyl complexes’ Re(CO)_5_Br [30], *fac*-[Et_4_N]_2_[Re(CO)_3_Br_3_] [31], and ^99m^Tc-tricarbonyl precursor [32] *fac*-[^99m^Tc(CO)_3_(H_2_O)_3_]^+^ was prepared according to the methods described in the literature. For the latter, a vial containing 4.4 mg of Na_2_CO_3_, 5.5 mg of NaBH_4_, and 12 mg of Na−K tartrate was purged with carbon monoxide gas for 1 min, and then a solution of Na^99m^TcO4 (0.5 mL, ∼370 MBq) was added. The vial was heated at 85 °C for 30 min. After it cooled, the pH was adjusted to 6 using 1 N HCl, and a sample was analyzed by HPLC (yield > 97%).

### 2.2. Synthesis of Rhenium Complexes

Rhenium complexes were synthesized using ciprofloxacin dithiocarbamate (Cip-DTC) as the bidentate ligand and various phosphines as monodentate ligands. The structures of the synthesized complexes were confirmed through IR and NMR spectroscopy.

#### 2.2.1. Synthesis of Complex *fac*-[Re(CO)_3_(Cip-DTC)(H_2_O)] (**1**)

A solution of Cip-DTC (90 mg, 0.2 mmol) in 2 mL of water was added to a stirred solution of *fac*-[Et_4_N]_2_[Re(CO)_3_Br_3_] (155 mg, 0.2 mmol) in 5 mL of water. The reaction mixture was stirred at 50 °C for 4 h. After cooling to room temperature, the yellow solid product was collected by filtration, washed with small amounts of water and methanol, and dried under vacuum. Yield: 80 mg (58%). IR (cm^−1^): 3408 (H_2_O), 2033, 2013, 1887 (*fac*-Re(CO)_3_), 1720, 1626 (C=O), 1241, 1011 (CS_2_). ^1^H NMR (500 MHz, DMSO-*d_6_*) δ_H_ (ppm) 8.63 (1H, H-12), 7.92 (1H, H-10), 7.58 (1H, H-7), 4.11 (2H, H-2/H-5), 3.44 (4H, H-3/H-4), 3.75 (2H, H-16), 1.33 (4H, H-17/H-18).

#### 2.2.2. Synthesis of *fac*-[Re(CO)_3_(Cip-DTC)(PPh_3_)] (**2**)

Method A: Two-Step Synthesis Using the Intermediate Complex **1**: To a stirred solution of the intermediate complex **1** (24 mg, 0.03 mmol) in 3 mL of methanol, triphenylphosphine (PPh_3_) (10.5 mg, 0.04 mmol) dissolved in 2 mL of methanol was added. The reaction mixture was stirred at 50 °C for 4 h. After cooling to room temperature, the yellowish solid product was collected by filtration, washed with water and methanol, and dried under vacuum. Yield: 17 mg (61%).

Method B: One-Pot Synthesis Using *fac*-[Et_4_N]_2_[Re(CO)_3_Br_3_]: Equimolar quantities of Cip-DTC and PPh_3_ (each 0.1 mmol) were added simultaneously to a solution of *fac*-[Et_4_N]_2_[Re(CO)_3_Br_3_] (77 mg, 0.1 mmol) in 10 mL of methanol. The mixture was refluxed for 2 h. After cooling to room temperature, the product was collected by filtration, washed with water and methanol, and dried under vacuum. Yield: 70 mg (74%).

Method C: One-Pot Synthesis Using Re(CO)_5_Br: Equimolar amounts of Re(CO)_5_Br (40.6 mg, 0.1 mmol), Cip-DTC (45.1 mg, 0.1 mmol), and PPh_3_ (26 mg, 0.1 mmol) were dissolved in 10 mL of methanol and stirred at 50 °C for 4 h. The product was collected by filtration, washed with water and methanol, and recrystallized. Yield: 77 mg (82%). IR (cm^−1^): 2015, 1884 (*fac*-Re(CO)_3_), 1618, 1584 (C=O), 1232, 1010 (CS_2_), 741, 692 (C-H, monosubstituted phenyl). ^1^H NMR (500 MHz, DMSO-*d_6_*) δ_H_ (ppm) 8.54 (1H, H-12), 7.81 (1H, H-10), 7.39 (1H, H-7), 3.60 (2H, H-2/H-5), 3.27 (4H, H-3/H-4), 3.65 (2H, H-16), 1.30–1.15 (4H, H-17/H-18), 7.51-7.05 (15H, PPh_3_).

#### 2.2.3. One-Pot Synthesis of Rhenium Complexes **3**–**6** Using Re(CO)_5_Br

Following the optimized method for complex **2**, rhenium (I) tricarbonyl complexes **3**–**6** were synthesized using a one-pot approach. Equimolar amounts (0.1 mmol each) of Re(CO)_5_Br, Cip-DTC and the respective phosphine ligand (TMPP, MePPh_2_, DMPP, or ADAP) were dissolved in 10 mL of methanol. The reaction mixtures were stirred at 50 °C for 4 h. After cooling to room temperature, the resulting solid products were collected by filtration, washed with water and methanol, and recrystallized to obtain pure complexes.

*fac*-[Re(CO)_3_(Cip-DTC)(TMPP)] (**3**): Yield: 62 mg (60%). IR (cm^−1^): 2011, 1912(sh), 1881 (*fac*-Re(CO)_3_), 1621, 1591 (C=O), 1251 (CH_3_O-Ph), 1234, 1011 (CS_2_), 801 (C-H, p-substituted phenyl). ^1^H NMR (500 MHz, DMSO-*d_6_*) δ_H_ (ppm) 8.53 (1H, H-12), 7.81 (1H, H-10), 7.37 (1H, H-7), 3.67 (2H, H-2/H-5), 3.34 (4H, H-3/H-4), 3.57 (2H, H-16), 1.30–1.02 (4H, H-17/H-18), 3.71 (9H, OCH3), 7.56-6.84 (12H, TMPP).

*fac*-[Re(CO)_3_(Cip-DTC)(MePPh_2_)] (**4**): Yield: 65 mg (74%). IR (cm^−1^): 2011, 1888 (*fac*-Re(CO)_3_), 1732, 1622, 1584 (C=O), 1232, 1011 (CS_2_), 736, 691 (C-H, monosubstituted phenyl). ^1^H NMR (500 MHz, DMSO-*d_6_*) δ_H_ (ppm) 8.60 (1H, H-12), 7.88 (1H, H-10), 7.48 (1H, H-7), 3.76 (2H, H-2/H-5), 3.39 (4H, H-3/H-4), 3.72 (2H, H-16), 1.32–1.02 (4H, H-17/H-18), 2.28 (3H, P-CH_3_), 7.56-7.05 (10H, PPh_2_Me).

*fac*-[Re(CO)_3_(Cip-DTC)(DMPP)] (**5**): Yield: 56 mg (69%). IR (cm^−1^): 2011, 1899 (*fac*-Re(CO)_3_), 1737, 1630 (C=O), 1232, 1012 (CS_2_), 745, 692 (C-H, monosubstituted phenyl). ^1^H NMR (500 MHz, DMSO-*d_6_*) δ_H_ (ppm) 8.59 (1H, H-12), 7.87 (1H, H-10), 7.48 (1H, H-7), 3.47 (2H, H-2/H-5), 3.43 (4H, H-3/H-4), 3.70 (2H, H-16), 1.32–1.00 (4H, H-17/H-18), 1.93 (6H, P-(CH_3_)_2_), 7.56-7.05 (10H, PPh_2_).

*fac*-[Re(CO)_3_(Cip-DTC)(ADAP)] (**6**): Yield: 58 mg (70%). IR (cm^−1^): 2016, 1912(sh), 1881 (*fac*-Re(CO)_3_), 3671, 2987, 2971, 2900 (CH_2_, adamantane), 1621, 1584 (C=O), 1232, 1012 (CS_2_). ^1^H NMR (500 MHz, DMSO-*d_6_*) δ_H_ (ppm) 8.55 (1H, H-12), 7.82 (1H, H-10), 7.44 (1H, H-7), 4.15 (2H, H-2/H-5), 3.45 (4H, H-3/H-4), 3.57 (2H, H-16), 1.27–1.00 (4H, H-17/H-18), 4.47 (6H, NCH_2_N), 4.20 (6H, PCH_2_N).

### 2.3. Preparation of Technetium-99m Complexes

Attention! All procedures involving the radioactive gamma emitter ^99m^Tc were conducted by trained personnel in a laboratory equipped with appropriate shielding and safety measures, in accordance with regulatory guidelines for radionuclide handling.

The preparation of technetium-99m complexes **1′**–**6′** involved the initial formation of the intermediate complex *fac*-[^99m^Tc(CO)_3_(Cip-DTC)(H_2_O)] (**1′**), followed by the substitution of the water molecule with various phosphine ligands to yield the final complexes *fac*-[^99m^Tc(CO)_3_(Cip-DTC)(P)] (**2′**–**6′**).

#### 2.3.1. Synthesis of Intermediate Complex *fac*-[^99m^Tc(CO)_3_(Cip-DTC)(H_2_O)] (**1′**)

To synthesize the intermediate complex **1′,** a solution of Cip-DTC (0.5 mg) in 0.5 mL of H_2_O was combined with 0.5 mL (~64 MBq) of *fac*-[^99m^Tc(CO)_3_(H_2_O)_3_]^+^ in a vial. The vial was sealed with a rubber stopper and an aluminum crimp cap and incubated at 70 °C for 30 min. After cooling, the sample was analyzed by HPLC, confirming the formation of a single radioactive complex with a more than 95% yield. The activity recovery after HPLC column injections was monitored and found to be quantitative. For the in vitro and in vivo evaluation, the final product was purified through HPLC and isolated with a radiochemical purity (RCP) of 99%. The identity of complex **1′** was established through comparative HPLC studies using the analogous, well-characterized rhenium complex **1** as a reference.

#### 2.3.2. Synthesis of *fac*-[^99m^Tc(CO)_3_(Cip-DTC)(P)] Complexes (**2′**–**6′**)

For the synthesis of complexes **2′**–**6′**, 0.2 mL of *fac*-[^99m^Tc(CO)_3_(Cip-DTC)(H_2_O)] (**7**) was transferred to a vial containing a solution of the respective phosphine ligand (PPh_3_, TMPP, MePPh_2_, DMPP, or ADAP) in 0.5 mL of MeOH. The vial was sealed with a rubber stopper and an aluminum crimp cap and incubated at 37 °C for 30 min. After cooling, the samples were analyzed by HPLC, which confirmed the formation of single radioactive complexes with a more than 95% yield. The activity recovery after HPLC column injections was quantitative. For the in vitro and in vivo evaluations, the final product was purified through HPLC and isolated with a radiochemical purity (RCP) of 99%. The identity of complexes **2′**–**6′** was established through comparative HPLC studies using the well-characterized analogous rhenium complexes **2**–**6** as reference materials.

### 2.4. Stability Studies for ^99m^Tc-Complexes

The preparation of the ^99m^Tc-labeled complexes from synthesis to HPLC purification was completed within 1.5 h. Then, the stability of HPLC purified technetium-99m complexes **1′**–**6**, with an RCP of 99% (based on HPLC), was assessed by monitoring their RCP over time and under various conditions using HPLC analysis based on established literature methods and is briefly described here [33].

#### 2.4.1. Stability in Reaction Mixture

The stability of complexes **1′**–**6′** in their reaction mixtures was monitored by periodic HPLC analysis over 6 h. Aliquots were taken at 1, 3, and 6 h and analyzed to observe any changes in the radio chromatograms.

#### 2.4.2. Stability of Purified Complexes

After purification by HPLC, the stability of the isolated technetium-99m complexes was evaluated over 6 h. The purified complexes were stored at room temperature, and aliquots were taken at 1, 3, and 6 h for HPLC analysis.

#### 2.4.3. Cysteine and Histidine Challenge Studies

Challenge experiments were performed by incubating the purified complexes with 10^−3^ M solutions of cysteine and histidine at 37 °C. For each complex (**1′**–**6′**), 0.2 mL of the purified complex was mixed with 0.2 mL of the cysteine or histidine solution and incubated for 6 h. Aliquots were taken at the end of the incubation period and analyzed by HPLC.

### 2.5. Lipophilicity Studies for ^99m^Tc-Complexes

The lipophilicity of HPLC purified technetium-99m complexes **1′**–**6′** was determined by measuring the partition coefficient (logD_7.4_) between *n*-octanol and phosphate-buffered saline (PBS, pH 7.4) based on established literature methods and is briefly described here. An aliquot of each technetium-99m complex was added to a mixture of equal volumes (2.0 mL each) of *n*-octanol and PBS (pH 7.4) in a 5 mL centrifuge tube. The mixture was vigorously vortexed for 2 min and then centrifuged at 5000 rpm for 5 min to ensure complete phase separation. After centrifugation, 0.1 mL aliquots from both the *n*-octanol and PBS phases were sampled and their radioactivity was measured using a gamma counter. The log P was calculated using the following equation: logD7.4 = (radioactivity in *n*-octanol/radioactivity in PBS). The procedure was repeated three times for each complex to ensure reproducibility.

### 2.6. Biodistribution Studies for ^99m^Tc-Complexes

All the biodistribution experiments were carried out in compliance with the national laws and European protocols (2010/63/EU) related to the conduct of animal experimentation. The in vivo behavior of HPLC purified technetium-99m complexes **1′**–**6′** was evaluated through biodistribution studies in Swiss Albino mice (40 ± 5 g) with induced infection and aseptic inflammation. The experiment was conducted in accordance with the guidelines for the protection of animals used for scientific purposes. Mice were divided into groups and prepared for biodistribution studies by inducing infection and aseptic inflammation. Twenty-four hours prior to the injection of the technetium complexes, the left thigh muscle of each mouse was inoculated with 50 µL of a Staphylococcus aureus suspension (1 × 10^6^ CFU/mL) to induce infection. Aseptic inflammation was induced in the right thigh muscle by injecting 50 µL of turpentine oil. The technetium-99m complexes were administered intravenously via the tail vein. Each mouse received an injection of 0.037 MBq of the respective technetium-99m complex. The biodistribution was studied at two different time points, 15 min and 120 min post-injection. At each time point, the mice were sacrificed under anesthesia. Blood samples were obtained by cardiac puncture. The organs of interest were collected after dissection, including the liver, heart, kidneys, stomach, intestines, spleen, muscle (normal, infected, and inflamed), lungs, pancreas, and urinary bladder. The organs were made free from adhering tissues and transferred into pre-weighed counting vials. The radioactivity of the collected samples was measured using a shielded well-type gamma counter. The results were expressed as a percentage of injected dose per gram of tissue (% ID/g). The radioactivity ratios in infected muscle to normal muscle and inflamed muscle to normal muscle were calculated to assess the specificity and selectivity of the technetium complexes for infection and inflammation.

## 3. Results and Discussion

### 3.1. Preparation of Rhenium Complexes

**Figure 1:**
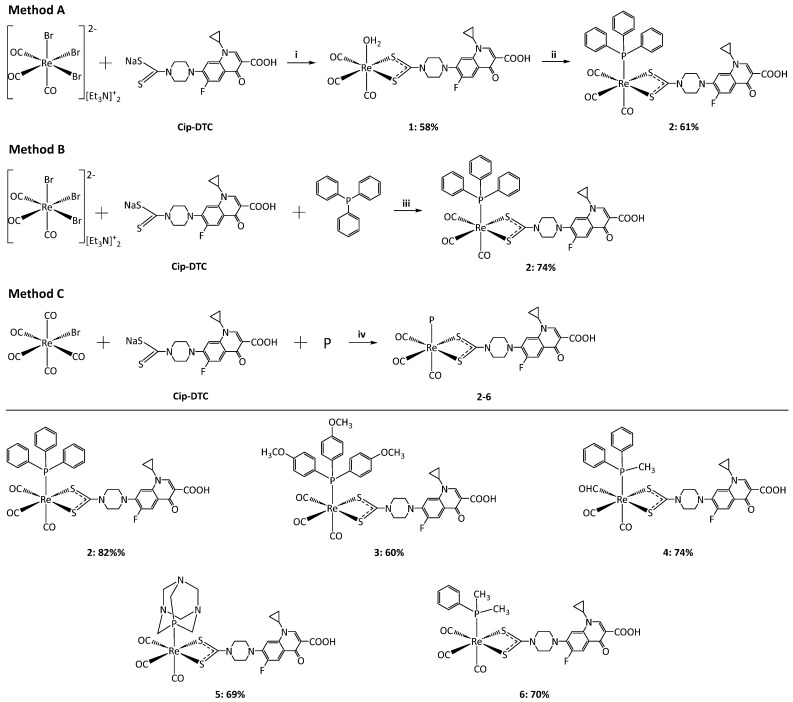
Synthesis of rhenium complexes **1**–**6**: (**i**) method A: H_2_O, 50 °C, 4 h; (**ii**) methanol, 50 °C, 4 h; (**iii**) method B: methanol, reflux, 2 h; (**iv**) method C: P (TMPP, MePPh2, DMPP, or ADAP), 50 °C, 4 h.

The synthesis of rhenium complexes **1**–**6** involved the use of ciprofloxacin dithiocarbamate derivatives (Cip-DTC) and various phosphines, exploiting the [2 + 1] strategy to produce neutral tricarbonyl complexes (Figure 1). In this approach, the ciprofloxacin derivative coordinates as a bidentate, while one phosphine coordinates to rhenium as a monodentate, providing stability and specificity to the resulting complexes. The structural integrity of the complexes was confirmed through IR and NMR spectroscopy, highlighting the successful coordination of the dithiocarbamate and phosphine ligands to the rhenium center.

Rhenium(I) tricarbonyl complex **2** was prepared using three different methods. Initially, complex **2** was synthesized from the intermediate dithiocarbamate complex **1**. Complex **1** was obtained by adding Cip-DTC to a solution of the rhenium tricarbonyl precursor [Et_4_N]_2_[Re(CO)_3_Br_3_] in water. This reaction led to the formation of intermediate complex **1**, which was then further reacted with monodentate PPh_3_, resulting in the substitution of the labile coordinated water molecule by PPh_3_. This method yielded complex **2** with a moderate yield of 58%. In a second method, complex **2** was synthesized by simultaneously adding equimolar quantities of Cip-DTC and PPh_3_ to a solution of the rhenium tricarbonyl precursor in methanol. This one-step approach provided a higher yield of 74%, suggesting a more efficient interaction between the ligands and the rhenium precursor. The most efficient synthesis was achieved through a one-pot reaction using Re(CO)_5_Br, where equimolar amounts of Re(CO)_5_Br, Cip-DTC, and PPh_3_ were stirred at 50 °C for 4 h. This approach, method C, produced pure complex **2** in a high yield of 82%, reducing the synthetic steps compared to those of method B.

Following the optimized one-pot synthesis approach for complex **2**, Re-tricarbonyl complexes **3**–**6** were prepared similarly. Equimolar amounts of Re(CO)_5_Br, Cip-DTC, and the respective phosphines (TMPP, MePPh_2_, DMPP, or ADAP) were stirred at 50 °C in methanol for 4 h. The reactions yielded pure solid compounds, which were subsequently recrystallized to afford complexes **3**–**6** in good yields (60–74%). This method consistently produced high yields across different phosphine ligands, demonstrating its versatility and efficiency.

The presence of the *fac*-[Re(CO)_3_]^+^ fragment in complexes **1**–**6** was confirmed by their characteristic CO stretching modes (2033–1887 cm^−1^) observed in the IR spectra [25,34]. For complex **1**, additional peaks at 3408 cm^−1^ indicated the presence of water, confirming the coordination environment of the intermediate complex. In complexes **2**–**6**, other characteristic absorptions corresponding to their respective structures were observed. The CO stretching modes of the pyridone and carboxyl groups of ciprofloxacin were observed at 1629–1620 cm^−1^ and 1709 or 1578 cm^−1^, respectively. These peaks confirmed the incorporation of the ciprofloxacin moiety. Peaks at 1240–1238 cm^−1^ and 1011–1006 cm^−1^ due to C-S stretching modes confirmed the coordination of the dithiocarbamate group. Complexes **2**, **4**, and **5** exhibited characteristic peaks at 742–743 cm^−1^ and 691–693 cm^−1^ due to the C–H stretching vibrations of the monosubstituted phenyl rings of the phosphines, indicating the successful coordination of the phosphine ligands [35]. Additionally, the IR spectrum of complex **3** displayed a peak at 801 cm^−1^ due to the C–H stretching vibrations of the *p*-substituted phenyl ring and a peak at 1251 cm^−1^, indicative of the C–O–C stretching vibration of the methoxy group, confirming the incorporation of the methoxy-substituted phosphine.

The ^1^H NMR spectra of all complexes were recorded, revealing the expected number of signals and confirming the structural integrity of the synthesized compounds. Notably, the aromatic protons of the ciprofloxacin moiety did not shift, indicating that coordination did not occur through the carbonyl or carboxyl groups, which remained uncoordinated. Significant chemical shifts were observed for the protons of the piperazine group, especially those near the carbamate group, suggesting coordination through the sulfur atoms of the dithiocarbamate moiety. Signals corresponding to the protons of the monodentate ligands were also observed, further confirming their successful coordination. The ^1^H NMR chemical shifts for complexes **1**–**6**, as well as for ciprofloxacin and the ligand Cip-DTC, are summarized in Table 1.

### 3.2. Preparation of ^99m^Tc-Complexes

**Figure 2:**
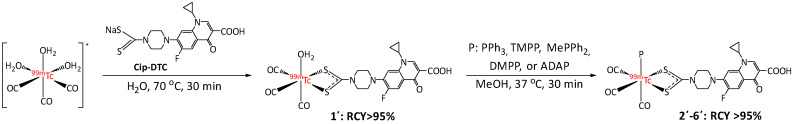
Radiosynthesis of technetium-99m complexes **1′**–**6′**.

**Figure 3:**
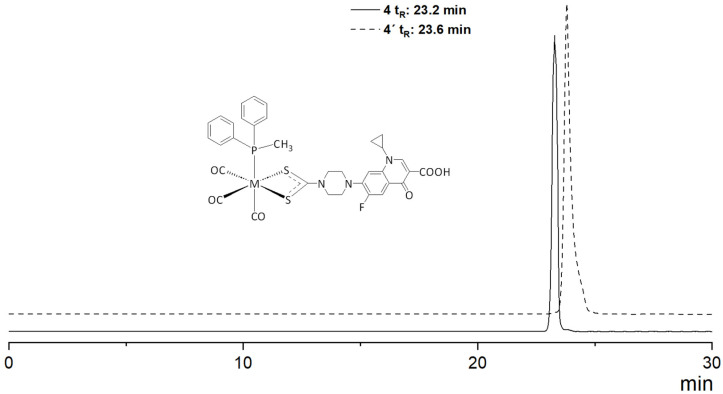
Representative comparative reverse-phase HPLC chromatograms: UV detection at 254 nm of complex **4** (solid). radiometric detection of complex **4′** (dash).

The preparation of homologous technetium-99m complexes **1′**–**6′** was achieved via ligand substitution using the precursor *fac*-[^99m^Tc(CO)_3_(H_2_O)_3_]^+^ [32] (Figure 2). The labile water molecules in this precursor were readily replaced by more stable and inert ligands, facilitating the formation of the desired complexes. The synthesis of complexes **2′**–**6′** followed a two-step protocol. Initially, intermediate complex **1′,** bearing the ligand Cip-DTC and a labile water molecule in the sixth position, was prepared. Subsequent additions of different phosphine ligands to complex **1′** and the replacement of the aqua molecule resulted in the formation of complexes **2′**–**6′.** These labeling reactions were conducted under consistent conditions regarding pH, temperature, reaction time, and reagent concentrations to ensure a standardized protocol. Optimal results were obtained by reacting the mixture of complex **1′** and phosphine ligands at 37 °C for 30 min. An HPLC analysis confirmed the successful formation of the complexes. The identities of these complexes were established by comparing their retention times to those of the analogous, well-characterized rhenium complexes (**1**–**6**) using parallel photometric and radiometric detection (Figure 3). The retention times (t_R_) for the ^99m^Tc complexes (**1′**–**6′)** and their rhenium analogs (**1**–**6**) were consistent, as shown in Table 2. This consistency confirmed the successful synthesis and structural integrity of the technetium complexes, aligning with the established rhenium coordination system.

### 3.3. Stability Studies

The stability of ^99m^Tc complexes **1′**–**6′** was evaluated through periodic HPLC analysis over 24 h. In the reaction mixtures, no significant changes were observed in the radiochromatograms, indicating high stability. After purification, the stability of the complexes remained high, with all complexes maintaining over 95% integrity. Specifically, complex **1′** showed 92% stability post-purification, while complexes **2′**–**6′** exhibited stability values ranging from 97% to 98%.

To assess the stability against transchelation, purified complexes **1′**–**6′** were incubated with 10^−3^ M histidine and cysteine at 37 °C. These complexes remained nearly intact after 6 h, with stability ranging from 96% to 98%. Complex **1′** also demonstrated good stability, maintaining 90% in cysteine and 91% in histidine. The results indicated that the ancillary ligands, particularly in complexes **2′**–**6′**, provided robust protection against potential degradation, ensuring their effectiveness in vivo for diagnostic applications.

### 3.4. Lipophilicity Studies

The lipophilicity of ^99m^Tc complexes **1′**–**6′**, expressed as logD_7.4_ values, is detailed in Table 2. This parameter is pivotal in influencing biodistribution and cellular uptake. Complex 1′ exhibited a logD_7.4_ value of 1.20, indicating relatively low lipophilicity. In contrast, complexes **2′** and **3′** showed higher logD_7.4_ values of 2.3 and 2.7, respectively, indicating increased lipophilicity, which enhances membrane permeability and tissue accumulation. Complexes **4′**–**6′**, with logD_7.4_ values ranging from 1.6 to 2.0, demonstrated moderate lipophilicity, suggesting a balanced distribution between aqueous and lipid environments, which supports effective targeting while maintaining sufficient clearance. These results indicate that complexes **2′** and **3′** may provide enhanced tissue penetration and prolonged circulation, which are beneficial for infection targeting, while the moderate lipophilicity of complexes **4′**–**6′** offers a favorable balance for diagnostic imaging applications.

### 3.5. Biodistribution Studies

The in vivo behavior of ^99m^Tc complexes **1′**–**6′** was evaluated in Swiss Albino mice with induced infection (Staphylococcus aureus) and aseptic inflammation (turpentine injection). Biodistribution studies were conducted at 15 and 120 min post-intravenous-injection, with the complexes purified by HPLC to remove excess unlabeled ligands and tracers of radiochemical impurities. The results, expressed as percent injected dose per gram of tissue (% ID/g), reveal distinct biodistribution patterns for each complex, influenced by their molecular structures, lipophilicity, and size (Table 3 and Table 4).

Studies have shown that complexes **1′**–**6′** have no significant uptake in the stomach at 2 h post-injection, indicating that the compounds do not re-oxidize to pertechnetate and maintain in vivo stability. Notably, complexes **1′** and **4′**–**6′** exhibited slow blood clearance within 120 min, while complexes **2′** and **3′** showed relatively stable or increasing blood radioactivity, respectively.

Complex **2′** demonstrated relatively stable blood radioactivity, 4.51 ± 1.32% ID/g at 15 min to 5.19 ± 0.81% ID/g at 120 min, with a high liver uptake of 32.36 ± 3.58% ID/g at 15 min and 27.16 ± 4.02% ID/g at 120 min with limited excretion to the intestines. In contrast, complex **3′** showed an increase in blood radioactivity from 2.54 ± 0.25% ID/g at 15 min to 6.71 ± 0.50% ID/g at 120 min, with a significant liver uptake at 47.48 ± 1.30% ID/g at 15 min and 27.58 ± 0.66% ID/g at 120 min with limited excretion to the intestines. This is consistent with findings reported in the literature for similar lipophilic ^99m^Tc-ciprofloxacin derivatives. For instance, the ^99m^Tc(CO)_3_–CPFXDTC complex has been found to have a liver uptake of 30.01% ID/g at 4 h p.i [23]. Additionally, other derivatives, such as ^99m^Tc(CO)_3_(L1) and ^99m^Tc(CO)_3_(L2), showed liver uptake values of 18.7% and 18.9% ID/g at 4 h, respectively [11]. These observations suggest that significant liver accumulation is common among ^99m^Tc-ciprofloxacin complexes, which could be attributed to their lipophilicity and the hepatobiliary excretion pathway. The less lipophilic complexes **4′**–**6′** demonstrated faster blood clearance, compared to **2′** and **3′**, and excretion to the intestines at 120 min p.i (7.97 ± 0.12–10.24 ± 1.11% ID/g).

The highest radioactivity uptake in the infected muscle was observed with complex **2′** at both time points, with 2.93 ± 0.09% ID/g at 15 min and 3.12 ± 0.14% ID/g at 120 min. Complex **3′** exhibited lower initial uptake in infected muscle at 0.27 ± 0.11% ID/g at 15 min but showed a substantial increase to 1.14 ± 0.05% ID/g at 120 min. In normal muscle, complex **2′** uptake decreased from 0.90 ± 0.09% ID/g at 15 min to 0.68 ± 0.05% ID/g at 120 min, while complex **3′** maintained a consistent low uptake of 0.34 ± 0.02% ID/g at both time points. The infected/normal muscle ratios further illustrate the targeting efficiency of these complexes. Complex **2′** showed ratios of 3.29 at 15 min and 4.62 at 120 min, indicating strong specificity for infection over normal tissue. Complex **3′** exhibited ratios of 0.79 at 15 min and 3.32 at 120 min, suggesting delayed but effective targeting. The aseptic inflammation/normal muscle ratios for complex **2′** were 1.34 at 15 min and 1.28 at 120 min, indicating better differentiation between infection and inflammation than complex **3′**, which had ratios of 0.79 at 15 min and 1.51 at 120 min.

The high stability of the complexes correlated with effective in vivo targeting and retention, ensuring the complexes remained intact during imaging. The lipophilicity of the complexes also played a crucial role in their biodistribution. Complex **2′**, with a lipophilicity of 2.30 and a molecular weight of 752.79 g/mol, along with its triphenylphosphine ligand, contributed to prolonged circulation, enhanced liver uptake, and robust infection site targeting. Complex **3′**, with a lipophilicity of 2.70 and the highest molecular weight of 842.87 g/mol, along with its tris(4-methoxyphenyl)phosphine ligand, resulted in delayed clearance, gradual tissue accumulation, and prolonged imaging. Compared to the other complexes, the larger size and higher molecular weight of complexes **2′** and **3′** impacted their biodistribution by enhancing plasma protein binding, reducing renal clearance, and favoring hepatobiliary excretion. These factors collectively prolonged their presence in the bloodstream and improved tissue penetration. The prolonged circulation time of complex **2′**, due to its moderately high lipophilicity and significant molecular weight, allowed better targeting of infection sites, as evidenced by high infected/normal muscle ratios.

In contrast, complex **3′**, with its higher lipophilicity and molecular weight, showed delayed but sustained uptake in infected tissues, making it suitable for extended imaging periods. The structural differences between complexes **2′** and **3′**, primarily due to their ancillary ligands, led to distinct biodistribution and clearance profiles. Both complexes exhibited high stability and the effective targeting of infection sites. Complex **2′** showed better early targeting efficiency and higher infected/normal muscle ratios, making it suitable for early imaging. Complex **3′**, with its higher lipophilicity, demonstrated delayed but substantial uptake in infection sites, which could be advantageous for prolonged imaging windows. Optimizing the balance between molecular weight, lipophilicity, and stability is crucial for the development of effective radiopharmaceuticals for diagnostic imaging.

Further studies focusing on refining these parameters can lead to the development of more effective radiopharmaceuticals with improved diagnostic specificity and clinical utility. A speculative but intriguing possibility is that the favorable results from complex **3′** may be partly due to in vivo metabolism involving the demethylation of the methoxy groups. If complex **3′** undergoes demethylation in the liver, the resulting hydroxyphenyl complexes probably reenter the blood circulation and accumulate in infected tissues. This hypothesis warrants further investigation through metabolic studies to confirm whether such demethylation occurs and to what extent it impacts the biodistribution and effectiveness of complex **3′**.

## 4. Conclusions

In this study, we successfully synthesized and characterized a series of [2 + 1] mixed-ligand ^99m^Tc-labeled tricarbonyl complexes using ciprofloxacin dithiocarbamate (Cip-DTC) and various phosphine ligands. The novel complexes demonstrated high stability, specificity, and effective targeting properties. The rhenium analogs served as structural models, ensuring that analogous technetium complexes displayed similar coordination parameters and physical properties, thus facilitating structural characterization. Stability studies indicated that the complexes maintained high integrity under various conditions, including challenges with cysteine and histidine. Lipophilicity studies revealed that complexes with higher logD_7.4_ values exhibited enhanced tissue penetration and prolonged circulation, which benefited infection targeting. Biodistribution studies in Swiss Albino mice with induced infection and aseptic inflammation showed distinct patterns influenced by molecular structure, lipophilicity, and size, with specific complexes demonstrating strong specificity for infection sites over normal tissues. These findings underscore the potential of these ^99m^Tc-labeled complexes as effective radiopharmaceuticals for the differential diagnosis of bacterial infections, advancing the field of nuclear medicine diagnostics. Future studies focusing on optimizing molecular weight, lipophilicity, and stability are warranted to further enhance the diagnostic specificity and clinical utility of these radiopharmaceuticals.

## Figures and Tables

**Table 1:** ^1^H NMR chemical shifts (ppm) for complexes **1**–**6**, as well as for ciprofloxacin (Cip) and ligand Cip-DTC, in DMSO-d_6_ at 25 °C.

Position	Cip	Cip-DTC	1	2	3	4	5	6
H-2/H-5	2.90	4.57	4.11	3.60	3.67 3.58	3.76 3.47	3.85	4.15 3.99
H-3/H4	3.24	3.17	3.44	3.27 2.89	3.34 2.80	3.39 3.09	3.43 3.19	3.45 3.32
H-7	7.54	7.42	7.58	7.39	7.37	7.48	7.48	7.44
H-10	7.90	7.80	7.92	7.81	7.81	7.88	7.87	7.82
H-12	8.66	8.54	8.63	8.54	8.53	8.60	8.59	8.55
H-16	3.83	3.61	3.75	3.65	3.57	3.72	3.70	3.57
H-17/H-18	1.31 1.18	1.28 1.00	1.33 1.15	1.30 1.04	1.30 1.02	1.32 1.11	1.32 1.08	1.27 1.00
P–Ph_3_				7.51 7.44	7.32 7.28 7.05 6.84	7.56 7.48	7.56 7.47 7.40	
P–CH_3_						2.28	1.93	
O–CH_3_					3.71			
NCH_2_N								4.47
PCH_2_N								4.20

**Table 2:** HPLC Retention times (t_R_) of rhenium and technetium-99m complexes. LogD_7.4_ of technetium-99m complexes. ^1^

Compound	M: Re	M: ^99m^Tc	
	(t_R_ min)	(t_R_ min)	LogD_7.4_
*fac*-[M(CO)_3_(Cip-DTC)(H_2_O)] (**1**, **1′**)	16.8	17.1	1.2
*fac*-[M(CO)_3_(Cip-DTC)(PPh_3_)] (**2**, **2′**)	26.1	26.4	2.3
*fac*-[M(CO)_3_(Cip-DTC)(TMPP)] (**3**, **3′**)	27.1	27.5	2.7
*fac*-[M(CO)_3_(Cip-DTC)(MePPh_2_)] (**4**, **4′**)	23.2	23.6	1.8
*fac*-[M(CO)_3_(Cip-DTC)(DMPP)] (**5**, **5′**)	22.3	22.7	2.0
*fac*-[M(CO)_3_(Cip-DTC)(ADAP)] (**6**, **6′**)	17.1	17.3	1.6

^1^ The values are the mean ± SD of three independent determinations.

**Table 3:** Tissue distribution of radioactivity (%ID/g) after injection of complexes **1′**–**3′** in mice. ^1^

Tissue	1′		2′		3′	
	15 min	120 min	15 min	120 min	15 min	120 min
Blood	14.07 ± 0.83	7.76 ± 0.29	4.51 ± 1.32	5.19 ± 0.81	2.54 ± 0.25	6.71 ± 0.50
Liver	22.05 ± 1.40	25.96 ± 2.23	32.36 ± 3.58	27.16 ± 4.02	47.48 ± 1.30	27.58 ± 0.66
Heart	1.93 ± 0.23	1.58 ± 0.21	1.23 ± 0.36	3.97 ± 0.25	0.74 ± 0.32	3.48 ± 0.23
Kidneys	5.16 ± 0.32	5.76 ± 0.65	1.30 ± 0.13	6.50 ± 0.36	1.40 ± 0.57	5.65 ± 0.78
Stomach	0.91 ± 0.20	1.42 ± 0.30	0.55 ± 0.23	1.23 ± 0.14	0.64 ± 0.61	1.21 ± 0.32
Intestines	0.71 ± 0.11	1.81 ± 0.15	0.14 ± 0.03	1.08 ± 0.07	0.36 ± 0.25	3.52 ± 0.19
Spleen	9.79 ± 1.69	5.24 ± 0.45	38.44 ± 3.92	37.02 ± 4.43	33.88 ± 2.81	23.10 ± 1.74
Muscle	0.18 ± 0.02	0.18 ± 0.01	0.90 ± 0.09	0.68 ± 0.05	0.13 ± 0.05	0.34 ± 0.02
Lungs	6.41 ± 1.14	4.33 ± 0.41	6.66 ± 1.45	10.20 ± 0.12	9.58 ± 0.86	8.67 ± 0.72
Pancreas	0.91 ± 0.04	0.82 ± 0.06	0.47 ± 0.20	1.04 ± 0.04	0.29 ± 0.13	0.93 ± 0.20
Infection	1.06 ± 0.20	1.80 ± 0.39	2.93 ± 0.09	3.12 ± 0.14	0.27 ± 0.11	1.14 ± 0.05
Aseptic Infl.	0.78 ± 0.51	1.33 ± 0.09	1.21 ± 0.07	0.86 ± 0.10	0.15 ± 0.07	0.52 ± 0.23
Urine *	0.15 ± 0.08	1.57 ± 0.98	3.95 ± 1.30	4.04 ± 1.23	0.00 ± 0.00	0.01 ± 0.02

^1^ All values are expressed as a percentage of the injected dose per gram of tissue weight (%ID/g), except for Urine *, for which values are expressed as a percentage of the injected dose per sample (%ID). All values are presented as mean ± standard deviation (SD).

**Table 4:** Tissue distribution of radioactivity (%ID/g) after injection of complexes **4′**–**6′** in mice. ^1^

Tissue	4′		5′		6′	
	15 min	120 min	15 min	120 min	15 min	120 min
Blood	3.20 ± 0.47	1.48 ± 0.11	0.91 ± 0.03	0.66 ± 0.05	2.18 ± 0.06	1.33 ± 0.10
Liver	42.11 ± 1.70	31.09 ± 0.72	35.51 ± 0.97	31.27 ± 5.71	20.95 ± 1.56	22.98 ± 2.70
Heart	4.36 ± 0.46	8.85 ± 0.63	2.98 ± 0.46	4.30 ± 1.47	4.27 ± 0.42	1.94 ± 0.31
Kidneys	5.92 ± 0.60	11.10 ± 0.70	3.20 ± 0.35	5.69 ± 1.44	8.05 ± 0.22	4.47 ± 0.52
Stomach	1.11 ± 0.50	2.23 ± 0.37	0.53 ± 0.22	2.33 ± 0.44	0.98 ± 0.29	3.14 ± 0.69
Intestines	1.27 ± 0.16	7.97 ± 0.12	0.61 ± 0.17	9.98 ± 1.22	3.22 ± 0.32	10.24 ± 1.11
Spleen	21.59 ± 0.98	8.14 ± 0.93	18.04 ± 0.73	7.64 ± 1.22	6.40 ± 0.66	2.55 ± 0.27
Muscle	0.56 ± 0.07	1.48 ± 0.05	0.40 ± 0.06	1.34 ± 0.09	1.01 ± 0.12	0.69 ± 0.05
Lungs	11.15 ± 0.19	9.41 ± 1.15	9.87 ± 0.89	5.37 ± 0.47	7.13 ± 0.35	3.06 ± 0.10
Pancreas	1.38 ± 0.21	3.21 ± 0.28	0.83 ± 0.18	3.68 ± 0.48	3.70 ± 0.26	2.27 ± 0.37
Infection	0.88 ± 0.15	2.83 ± 0.30	0.94 ± 0.31	3.10 ± 0.46	0.94 ± 0.09	0.69 ± 0.09
Aseptic Infl.	0.77 ± 0.12	1.88 ± 0.26	0.51 ± 0.14	2.37 ± 0.34	1.10 ± 0.06	0.66 ± 0.10
Urine *	0.01 ± 0.00	0.04 ± 0.03	0.03 ± 0.01	0.32 ± 0.12	0.37 ± 0.08	1.49 ± 0.23

^1^ All values are expressed as a percentage of the injected dose per gram of tissue weight (%ID/g), except for Urine *, for which values are expressed as a percentage of the injected dose per sample (%ID). All values are presented as mean ± standard deviation (SD).

## Data Availability

The data presented in this study are available on request from the corresponding author.
